# Coupled Thermal-pH and Sulfur Gradients Shape Chloroflexota-Dominated Bacterial Communities at a Residual Xinjiang Coal-Fire Vent

**DOI:** 10.3390/microorganisms14102327

**Published:** 2026-10-12

**Authors:** Ming Zhong, Haiping Huang, Yanze Sun, Yafeng Li, Wanxin Hao, Shuo Huang, Hong Zhang

**Affiliations:** 1 School of Energy Resources, China University of Geosciences, Beijing 100083, China; mingz@email.cugb.edu.cn; 2 School of Geosciences, Yangtze University, Wuhan 430100, China; 3 Department of Earth, Energy, and Environment, University of Calgary, Calgary, AB T2N 1N4, Canada; 4 Research Institute of Oil and Gas Technology, Changqing Oilfield Company, PetroChina, Xi’an 710018, China; 5 National Engineering Laboratory for Exploration and Development of Low Permeability Oil and Gas Fields, Changqing Oilfield Company, PetroChina, Xi’an 710018, China; 6 Hubei Key Laboratory of Petroleum Geochemistry and Environment, Yangtze University, Wuhan 430100, China

**Keywords:** coal fire, gas vent, thermal gradient, Chloroflexota, bacterial diversity

## Abstract

Residual coal-fire gas vents form steep thermal and geochemical gradients that create sharply distinct microbial habitats over short distances. We compared the microbial communities of two microhabitats at a residual vent near Kuitun City, Xinjiang: BS1, a gas-emitting fracture at 64 °C, and BS2, adjacent cooler sediment at 43 °C. BS1 had pH 6.8 and total sulfur 5.86 g/kg, whereas BS2 had pH 8.4 and total sulfur 1.90 g/kg. Illumina sequencing of the 16S rRNA gene V3–V4 region yielded 52,327 and 64,212 merged read pairs retained for downstream analyses for BS1 and BS2, respectively. BS1 contained fewer observed amplicon sequence variants (ASVs; 524 versus 1366) and lower Shannon diversity (5.626 versus 6.588). Chloroflexota dominated both communities but was more abundant in BS1 (43.83% versus 36.33%), whereas BS2 contained larger Proteobacteria and Bacteroidota fractions. BS1 was enriched in *Pyrinomonas*, unclassified Chloroflexota, unclassified Anaerolineae, and *Thermorudis*, while BS2 was enriched in *Thermogemmatispora*, *Massilia*, and *Arthrobacter*. At this vent, the hot fracture therefore supported a lower-diversity, Chloroflexota-enriched community, whereas cooler sediment supported a richer and more compositionally diverse community. Together with the mineralogical observations, these results reveal sharp site-scale partitioning of bacterial assemblages across a coupled thermal-pH-sulfur gradient.

## 1. Introduction

Extreme environments characterized by high temperatures, reducing conditions, or toxic compounds provide natural laboratories for examining the limits of microbial life and the ecological and evolutionary processes that enable persistence under severe physicochemical stress [1]. Research on thermophilic microorganisms has traditionally centered on geologically driven systems such as hot springs and deep-sea hydrothermal vents, where thermal and geochemical gradients are primary structuring forces [2,3]. In contrast, thermal habitats generated by coal combustion represent secondary high-temperature ecosystems in which heat, reducing gases, combustion products, and mineral transformation interact to create complex microenvironments. Despite their global occurrence, the microbiology of coal-fire gas vents remains comparatively understudied [4].

Coal fires produce spatially heterogeneous surface habitats shaped by steep thermal and geochemical gradients. Under oxygen-limited, water-bearing conditions, coal combustion generates CO and H_2_, while vent gases and altered substrates may contain sulfur species and other potentially toxic constituents [4,5,6]. These conditions vary sharply over short distances, producing strong environmental filters between gas-emitting fractures, thermally altered mineral surfaces, and surrounding soils [4,7]. Thermal and geochemical parameters in coal-fire systems are tightly coupled, producing composite environmental gradients that shape microbial assembly through multiple interacting environmental filters.

Previous studies demonstrate that microbial responses to coal-fire disturbance are strong but context dependent. Along a natural CO_2_-vent gradient, bacterial richness decreased with increasing CO_2_ flux, and Chloroflexota (including *Thermogemmatispora* and *Ktedonobacter*) became dominant [8]. At the Centralia coal-mine fire, the hottest soils exhibited reduced diversity and highly divergent communities, whereas recovered soils converged toward reference states as temperatures declined [7]. Other coal-fire systems highlight the role of gas-derived electron donors and available electron acceptors: a 58 °C vent community in the Kuznetsk Basin was dominated by Ktedonobacteria capable of oxidizing H_2_ or CO [4], whereas burning lignite seams supported thermophilic sulfate-reducing Firmicutes, including *Desulfofundulus* [5]. These contrasting systems illustrate how extreme thermal regimes and gas-derived energy sources jointly structure microbial communities, underscoring the complex ecological processes that govern functional potential in high-temperature environments.

Here, we investigate microbial community structure at a residual coal-fire gas vent near Kuitun City, Xinjiang, following excavation and sediment covering associated with fire control. Gas-emitting fractures, secondary mineral deposits, and localized biofilms remained exposed. We compared the microbial communities of a hot fracture substrate (BS1, 64 °C) with those of adjacent cooler sediment (BS2, 43 °C) to characterize how extreme thermal conditions and associated geochemical gradients influence bacterial diversity and community composition. Our objectives were to (i) characterize bacterial diversity within the two microhabitats; (ii) compare bacterial community composition between the hot fracture and adjacent cooler sediment; (iii) place Chloroflexota-related ASVs in a phylogenetic context; and (iv) develop ecological hypotheses regarding microbial assembly processes in a non-geothermal high-temperature environment.

## 2. Materials and Methods

### 2.1. Study Site and Sampling

**Figure 1:**
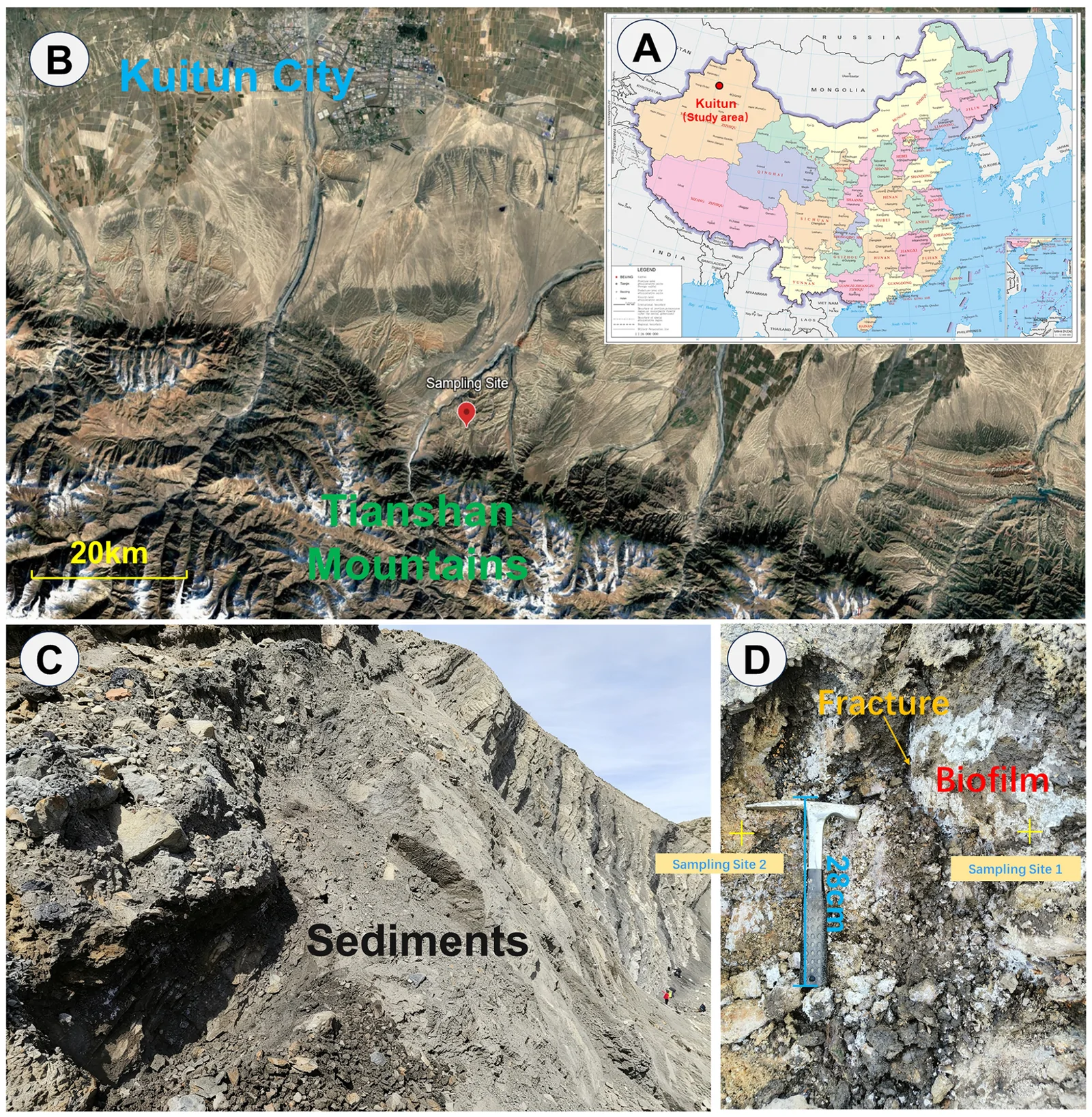
Geographical location and field sampling microhabitats. (**A**) Inset map showing the geographical position of the study area in Xinjiang, China. (**B**) Satellite imagery indicating the sampling site located at the northern foot of the Tianshan Mountains, south of Kuitun City (scale bar: 20 km). (**C**) Field photographic view of the exposed loose sediment slopes and surrounding terrain. (**D**) Detailed view of the sampling microhabitats displaying the hot fracture zone (Sampling Site 1) and neighboring cooler sediment (Sampling Site 2); macroscopically visible white film-like material occurred on both substrates and was the sampling target at BS1 (geological hammer provided for scale, length: 28 cm).

The sampling site was a naturally formed residual coal-fire gas vent associated with spontaneous subsurface coal-seam combustion on the northern piedmont of the Tianshan Mountains near Kuitun City, Xinjiang, northwestern China (44°00′43.87″ N, 85°02′05.53″ E; approximately 1840 m above sea level; Figure 1A). A coal-fire control project had previously used excavation and sediment covering at the site, leaving the original vents beneath loose sediment (Figure 1B). Removal of the surface sediment exposed gas-emitting fractures, secondary mineral precipitates, and white film-like material (Figure 1C).

Both samples were collected on 4 May 2026, with approximately 100 g of material obtained from each microhabitat. Sampling was purposive rather than random: the BS1 material was deliberately collected as white film-enriched sediment from the gas-emitting fracture surface, whereas the BS2 sample was taken from the adjacent cooler sediment, in which white film-like material was also present but was not the sampling target. Samples were sealed immediately, transported under refrigerated conditions, and stored at low temperature before DNA extraction.

### 2.2. DNA Sequencing and Sequence Processing

Total DNA was extracted from each substrate sample, and the bacterial 16S rRNA gene V3-V4 region was amplified using primers 338F (5′-ACTCCTACGGGAGGCAGCA-3′) and 806R (5′-GGACTACHVGGGTWTCTAAT-3′) [9,10]. Amplicon libraries were prepared and sequenced as paired-end reads on an Illumina NovaSeq 6000 platform (Illumina, Inc., San Diego, CA, USA). Each sample yielded three technical replicates processed in parallel [11].

Raw reads were quality-filtered with Trimmomatic v0.33 [12], and primer sequences were identified and removed using cutadapt v1.9.1 [13]. Denoising, paired-end merging, chimera removal, and amplicon sequence variant (ASV) inference were performed with DADA2 [14] implemented in QIIME 2 v2020.6 [15]. Features with fewer than three total reads were excluded. Taxonomic classification was performed using a combination of naive Bayes classification and BLAST v2.12.0+ (Basic Local Alignment Search Tool) searches against the SILVA 138 reference database [16].

### 2.3. Community Diversity and Taxonomic Profiles

Alpha diversity was assessed using multiple metrics, including observed ASVs, the abundance-based coverage estimator (ACE), Chao1, Simpson, Shannon, Faith’s phylogenetic diversity (PD), and Good’s coverage [17]. Relative-abundance patterns were examined at phylum and genus levels. For descriptive visualization, the six most abundant phyla were plotted individually, with all remaining phyla grouped as “Other.” Genera were selected to highlight major compositional contrasts between microhabitats and to emphasize Chloroflexota-related lineages relevant to the study objectives.

### 2.4. Phylogenetic Reconstruction

Chloroflexota-related ASV sequences and closely related reference sequences retrieved from GenBank were aligned using MAFFT v7.407 [18] (implemented in NGPhylogeny.fr, (https://ngphylogeny.fr/workflows/advanced/; accessed on 8 July 2026)). Poorly aligned or highly variable regions were automatically curated using BMGE v1.1 [19]. Maximum-likelihood (ML) phylogenetic trees were reconstructed using PhyML v3.3 [20] under the GTR + G + I substitution model, selected by Smart Model Selection (SMS) [21]. Branch support was evaluated with 1000 bootstrap replicates. The resulting phylogenetic tree was visualized and annotated using iTOL v6 [22].

### 2.5. Physicochemical and Mineralogical Analyses

Soil pH was determined in a 1:2.5 (*w*/*v*) soil-to-water suspension using a Sartorius PB-10 pH meter (Sartorius AG, Göttingen, Germany). Air-dried samples were passed through a 2 mm sieve, and 10.00 g was transferred to a 50 mL beaker and mixed with 25 mL of carbon-dioxide-free distilled water (self-prepared).

Total sulfur was measured by high-temperature catalytic combustion with oxygen and helium carrier gas on a Haineng E500 elemental analyzer (Haineng, Beijing, China) equipped with thermal-conductivity detection. Samples were wrapped in tin foil, introduced into the analyzer and read after approximately 10 min. Sulfur was oxidized to SO2 and reported on a dry-mass basis as g/kg after moisture correction. The calculation used was total sulfur (g/kg) = 10 × C_S_/(1 − k), where C_S_ is the instrument sulfur concentration and k is the moisture fraction.

Selected white sediment-associated material was examined by scanning electron microscopy (SEM, Thermo Fisher Scientific, Waltham, MA, USA)), and local elemental composition was measured by energy-dispersive X-ray spectroscopy (EDS, Bruker, Billerica, MA, USA) at an accelerating voltage of 15 kV. EDS values describe the marked local regions, whereas bulk X-ray diffraction (XRD) represents the mineralogical context.

Bulk mineralogical composition of sample BS1 was determined by X-ray diffraction using a PANalytical X’Pert-PRO diffractometer(Malvern Panalytical Ltd., Almelo, The Netherlands) equipped with an Empyrean Cu LFF HR tube and a Cu anode (Cu Kα1 radiation, λ = 1.5406 Å). The instrument was operated at 40 kV and 40 mA. Continuous scan data were collected over a 2θ range of 5.0° to 45.0° with a step size of approximately 0.013° and a counting time of 13.77 s per step.

## 3. Results

### 3.1. Sequencing Output and Coverage

Raw sequencing reads totaled 58,616 for BS1 and 80,009 for BS2. Following quality filtering, denoising, paired-end read merging and chimera removal, 52,846 and 66,132 non-chimeric merged read pairs were obtained for BS1 and BS2. After filtering low-abundance features, 52,327 (BS1) and 64,212 (BS2) merged read pairs were retained for downstream analyses. The average read lengths were 412.4 bp (BS1) and 416.4 bp (BS2). Good’s coverage reached 0.9998 for BS1 and 1.0000 for BS2, indicating that the lower richness observed in BS1 was not an artifact of incomplete sequencing sampling.

### 3.2. Within-Sample Diversity

The hot-fracture sample BS1 (64 °C) contained 524 observed ASVs, compared with 1366 in the cooler sediment sample BS2 (43 °C; Table 1). Richness estimators showed the same contrast: ACE was 525.71 for BS1 and 1366.42 for BS2, while Chao1 was 533.33 and 1366.25, respectively. BS1 also had a lower Shannon index than BS2 (5.6257 versus 6.5884; Table 1). Simpson values were similar but slightly lower in BS1 (0.9418 versus 0.9455), and Faith’s PD was 15.5614 in BS1 and 17.9561 in BS2. BS1 therefore showed lower richness, Shannon diversity and phylogenetic diversity than BS2. The complete set of alpha-diversity metrics is presented in Table 1.

**Table 1:** Alpha-diversity metrics of bacterial communities in BS1 (hot fracture, 64 °C) and BS2 (cooler sediment, 43 °C). All metrics were computed with QIIME 2 v2020.6 [15].

Metric	BS1 (Hot Fracture, 64 °C)	BS2 (Cooler Sediment, 43 °C)
Observed ASVs	524	1366
ACE	525.71	1366.42
Chao1	533.33	1366.25
Shannon index	5.6257	6.5884
Simpson index	0.9418	0.9455
Faith’s phylogenetic diversity	15.5614	17.9561
Good’s coverage	0.9998	1.0000

### 3.3. Phylum- and Genus-Level Composition

**Figure 2:**
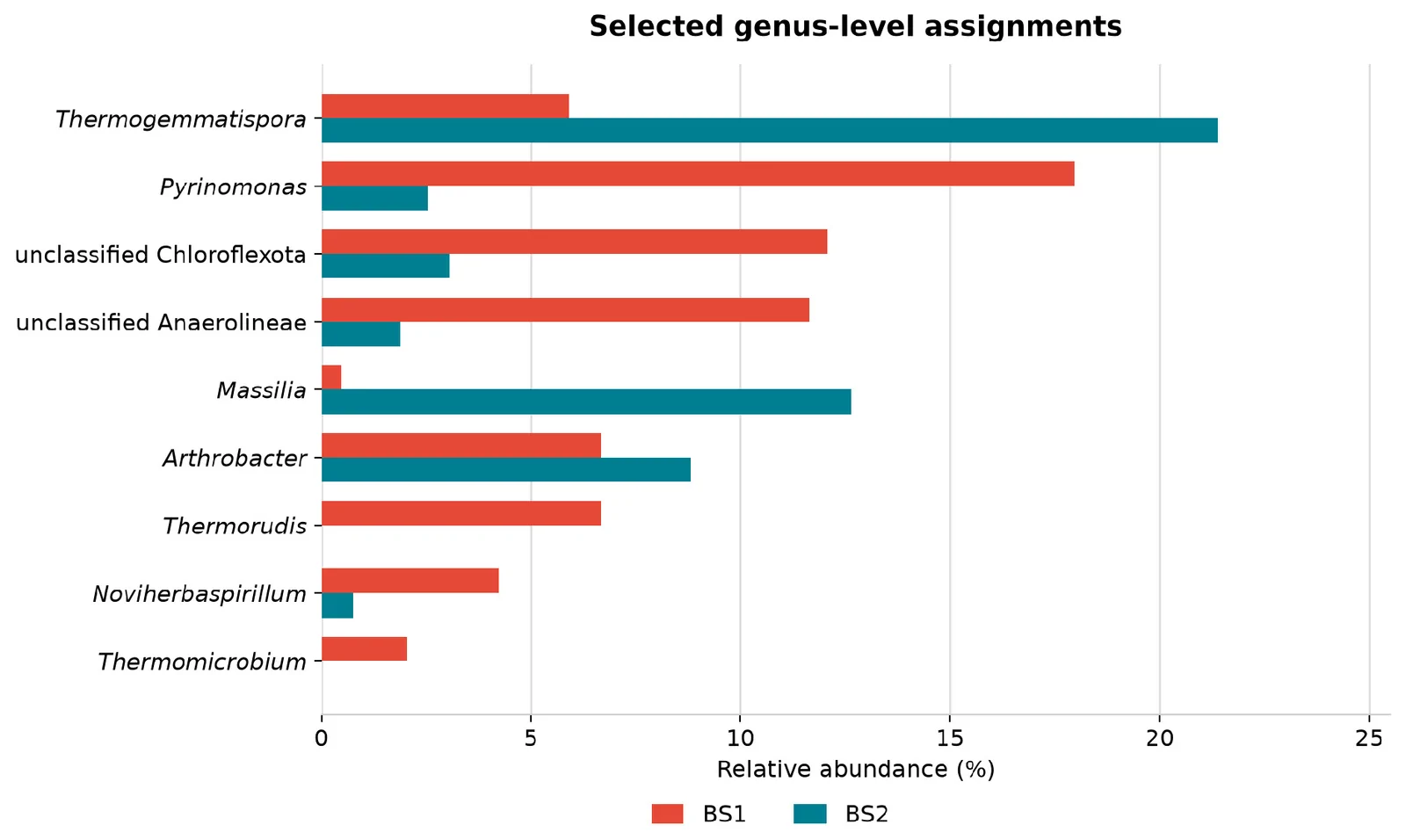
Relative abundances of selected bacterial genera showing the major compositional contrasts between BS1 (hot fracture, 64 °C) and BS2 (cooler sediment, 43 °C).

**Figure 3:**
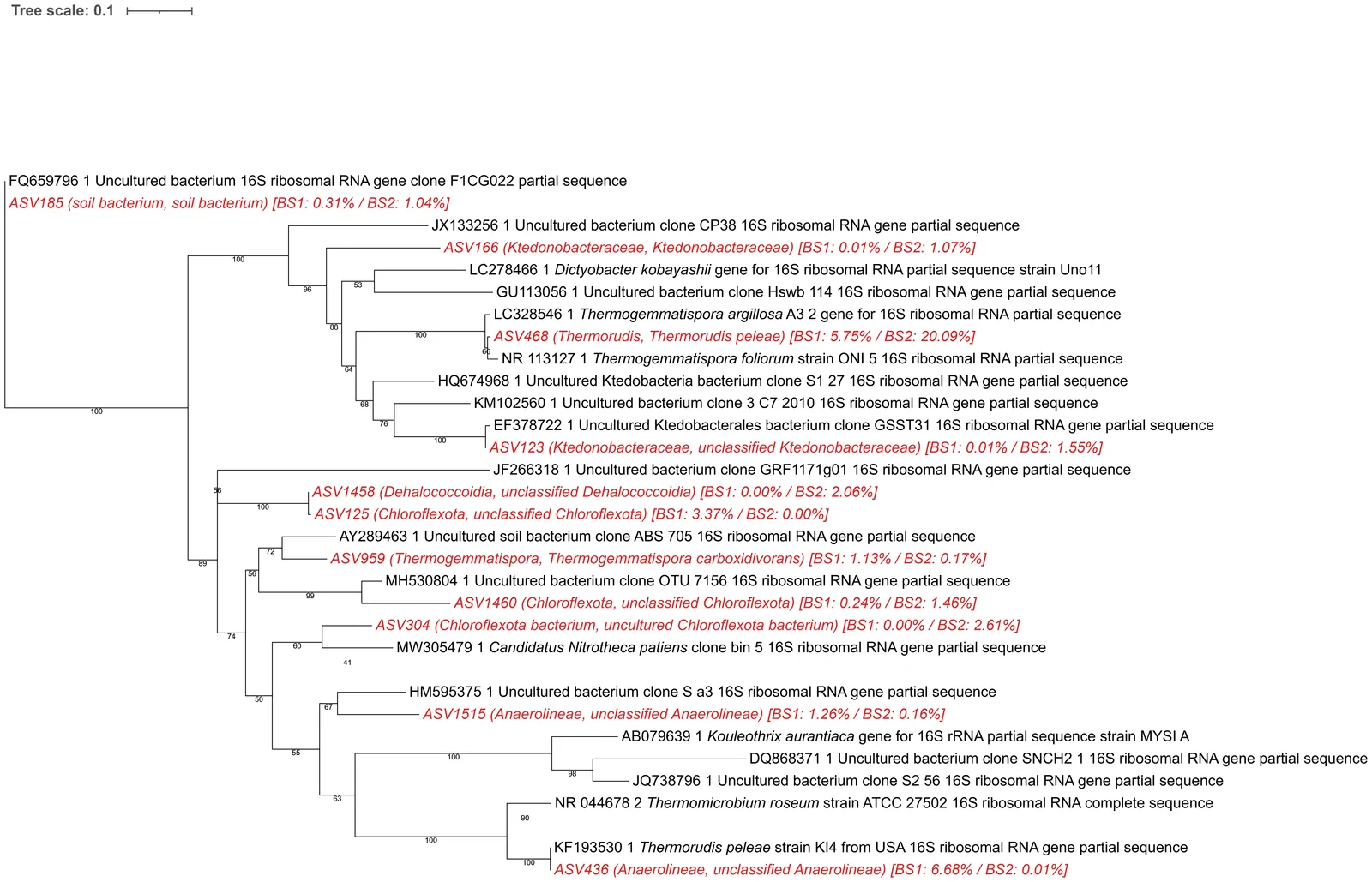
Maximum-likelihood phylogenetic tree of selected Chloroflexota-related ASVs (highlighted in red) and closely related reference sequences. The tree was constructed using the MAFFT–BMGE–PhyML pipeline on the NGPhylogeny.fr platform under the GTR + G + I model with 1000 bootstrap replicates. Bootstrap support values greater than 50% are displayed at nodes. The scale bar denotes 0.1 substitutions per site. Relative abundances of the selected ASVs in BS1 (64 °C) and BS2 (43 °C) are indicated in parentheses following the ASV IDs.

Chloroflexota was the most abundant phylum in both samples but represented a larger fraction of BS1 than BS2 (43.83% versus 36.33%). At the genus level, selected genera exhibited contrasting relative abundances between the two microhabitats (Figure 2). These phylum-level proportions were calculated by summing the relative abundances of all Chloroflexota-assigned ASVs in each sample. Figure 3 shows only the selected Chloroflexota-related ASVs used for phylogenetic placement, rather than the complete set of Chloroflexota ASVs; consequently, the percentages printed beside the ASV labels are not expected to sum to the phylum-level values. BS1 also contained Acidobacteriota (19.07%), Actinobacteriota (12.23%), Proteobacteria (11.90%), Firmicutes (4.32%), and Bacteroidota (1.05%). BS2 contained a larger Proteobacteria fraction (29.96%) and more Bacteroidota (6.50%), together with Actinobacteriota (12.36%), Acidobacteriota (5.90%), and Firmicutes (2.50%). Chloroflexota was selected for detailed phylogenetic analysis not simply because it was the dominant phylum, but because it was abundant in both microhabitats, relatively enriched in the hotter fracture, and included thermophilic lineages reported from coal-fire gas vents [4,23]. The two samples were not pooled for community-level analysis; Chloroflexota-related ASVs recovered from each microhabitat were instead compared within a common phylogenetic framework. A substantial fraction of the recovered ASVs could not be assigned to named genera (e.g., “unclassified Chloroflexota”, “unclassified Anaerolineae”), reflecting the sparse cultivation coverage of thermophilic lineages. Reference sequences labeled as “uncultured bacterium” were retained only to provide phylogenetic context when no more specific taxonomic assignment was available; these labels were not interpreted as evidence of novel taxa or specific metabolic capacities.

The higher relative abundance of *Thermogemmatispora* in BS2 is consistent with a comparatively moderate thermal niche. Reference strains such as Thermogemmatispora carboxidivorans show growth optima near 50–55 °C [24], which is closer to the temperature of BS2 (43 °C) than to that of BS1 (64 °C); the present data do not establish a causal temperature threshold. The two communities therefore differed in lineage-specific composition, not simply in the overall amount of thermophilic taxa [25].

### 3.4. Phylogenetic Placement of Chloroflexota-Related ASVs

Phylogenetic reconstruction placed the recovered ASVs into several distinct Chloroflexota-related lineages (Figure 3). ASV166 and ASV123 clustered within the Ktedonobacteraceae family, while ASV959 grouped with the genus *Thermogemmatispora* and the reference strain *T. carboxidivorans*. Additional ASVs were assigned to unclassified Chloroflexota or the class Anaerolineae. Furthermore, ASV468 was positioned near reference sequences of *Thermorudis peleae* and *Thermogemmatispora*.

### 3.5. Environmental Measurements, Micromorphology, and Bulk Mineralogy

**Figure 4:**
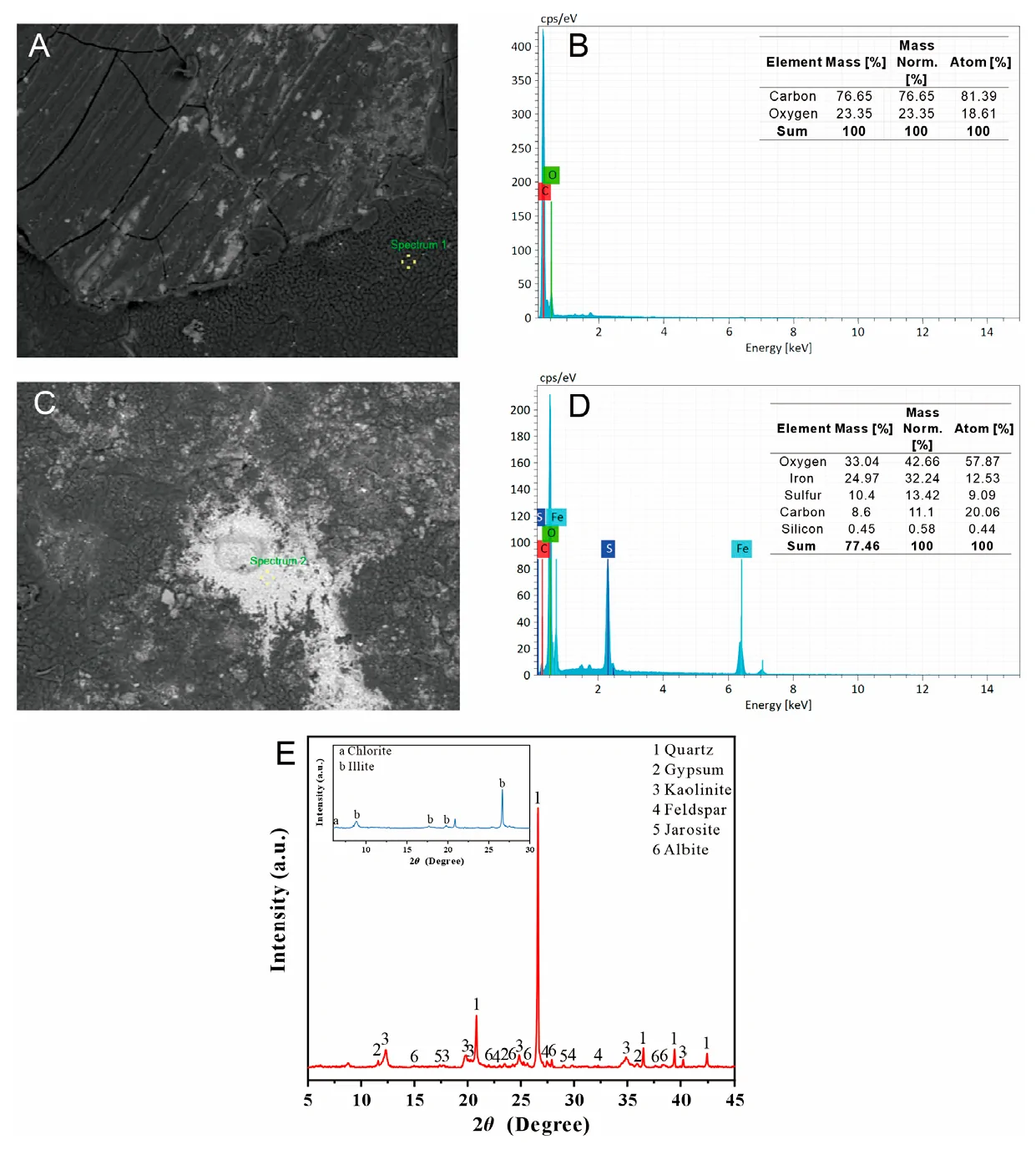
Micromorphology, local elemental composition and bulk mineralogy of white film-enriched sediment from BS1. (**A**,**C**) SEM micrographs showing wrinkled and reticulate film-like surface textures consistent with a biofilm-like surface structure. (**B**) Local EDS spectrum of the C/O-rich region, consistent with organic-rich biofilm-like material. (**D**) EDS spectrum of a distinct O-Fe-S-C-Si-enriched mineral region. (**E**) Whole-rock XRD pattern and quantitative mineral assemblage of BS1. EDS values describe the marked local regions, whereas XRD represents the bulk mineralogical context.

BS1 had a pH of 6.8 and total sulfur of 5.86 g/kg, while BS2 had a pH of 8.4 and total sulfur of 1.90 g/kg. SEM observations of the white film-enriched material from BS1 showed wrinkled to reticulate surface textures (Figure 4A,C), consistent with a biofilm-like surface structure. The selected C/O-rich region contained 76.65 wt% C and 23.35 wt% O (Figure 4B), a composition characteristic of organic matter that is distinct from the alunogen- and sulfate-dominated whitish mineral efflorescences reported at other coal-fire gas vents [4]. Because this film-enriched material was the sampling target for BS1 (Section 2.1), the SEM-EDS observations and the 16S rRNA gene data describe the same substrate. Chloroflexota, particularly the filamentous, spore-forming class Ktedonobacteria and the genus *Thermogemmatispora*, members of which have been isolated from geothermally heated biofilms [24], dominated the BS1 assemblage, consistent with an organic-rich, surface-associated microbial film. A spatially distinct bright region, by contrast, was enriched in normalized O (42.66%), Fe (32.24%), S (13.42%), C (11.10%) and Si (0.58%) (Figure 4D), representing an associated iron-sulfur mineral domain. Together, these observations reveal strong local chemical heterogeneity, with an organic-rich film intimately associated with secondary iron-sulfur mineral phases. We note that individual bacterial cells were not directly resolved at the magnifications used; higher-resolution electron microscopy or fluorescence in situ hybridization would be required to identify the cells in situ and to confirm their taxonomic identity.

Quantitative XRD analysis indicates that sample BS1 comprises a quartz-rich (58.3%) and clay-bearing (28.4%) matrix, accompanied by subordinate plagioclase (5.1%), K-feldspar (3.2%), gypsum (2.6%), and jarosite (2.4%) (Figure 4E). Consequently, BS1 represents a silica- and clay-dominated sediment with secondary sulfate and iron-bearing phases.

## 4. Discussion

### 4.1. Thermal-Geochemical Coupling Drives Microhabitat Divergence

The two adjacent microhabitats at the Xinjiang vent differ sharply in their microbial communities, and this divergence tracks tightly coupled thermal and geochemical gradients. The shift from BS1 to BS2 spans an integrated environmental cline in which elevated thermal stress (64 °C) operates together with localized acidity (pH 6.8), a higher sulfur burden and continuous gas advection; thermal, geochemical, and mineralogical parameters are tightly coupled, producing composite environmental gradients that influence microbial assembly through multiple interacting environmental filters. At BS1, persistent venting may reduce atmospheric oxygen penetration into the fracture interior, potentially creating a more reducing microenvironment than the adjacent sediment. The resulting oxygen-redox transition probably acts as a strong biophysical filter, replacing the highly restricted, stress-selected community at the vent with a more diverse, soil-derived assemblage.

The complementary alpha-diversity metrics indicate that the contrast between BS1 and BS2 involved both taxon richness and phylogenetic breadth. Observed ASVs, ACE and Chao1 were approximately 2.6-fold lower in BS1. The Shannon index was also lower, and the slightly lower Simpson value indicates reduced diversity; the relatively small difference in Simpson values suggests that dominance structure differed less strongly than richness. Faith’s PD was also lower in BS1, consistent with a narrower phylogenetic breadth. Good’s coverage values close to 1.0 for both samples indicate that the richness contrast was unlikely to result from unequal sequencing completeness. This pattern agrees with the reduced richness and Faith’s PD reported for fire-affected soils at the Centralia coal-mine fire [7] and with the decreases in Chao1 richness and Shannon diversity observed along a naturally occurring CO_2_-vent gradient [8].

Physical proximity across this fracture-sediment boundary does not erase functional or compositional segregation. The physicochemical environment drops steeply over a scale of centimeters, and advective gas shear imposes strong spatial dispersal limitation [23]. The more severe thermal and geochemical conditions at BS1 may constrain the establishment of many mesophilic taxa, while favoring a narrower subset of stress-tolerant lineages. This site-specific partitioning matches broader biogeographical patterns in the Centralia, Kuznetsk and Altai coal-fire systems. It confirms that intense environmental filtering predictably controls community assembly at active thermal vents [4,5,7,23,26].

### 4.2. Lineage-Specific Niche Partitioning Within Thermal Chloroflexota

The strong enrichment of Chloroflexota and associated thermophilic lineages at the hot fracture is consistent with microbial adaptations previously reported from coal-fire gas-vent environments. Previous studies of coal-fire systems have documented reduced gases such as CO and H_2_ and have identified thermophilic microbial lineages capable of oxidizing these compounds [4,6,23,24,27]. Such gases could therefore represent potential electron donors in residual coal-fire environments. The enrichment of *Pyrinomonas*, unclassified Chloroflexota, and Anaerolineae in BS1 is therefore interpreted as being consistent with, rather than direct evidence for, a gas-influenced thermophilic niche.

The response of Chloroflexota to venting stress is lineage-specific rather than phylum-wide [25]. Unclassified Anaerolineae and *Pyrinomonas* peak within the hotter, more acidic and sulfur-rich BS1 fracture. In contrast, *Thermogemmatispora* is comparatively enriched at the moderated thermal and mildly alkaline conditions of BS2 (43 °C, pH 8.4). Reference strains such as *T. carboxidivorans* show growth optima near 50–55 °C [24], which is closer to the temperature of BS2 than to that of BS1; the present data do not establish a causal upper temperature limit. This temperature contrast, combined with the lower sulfur load and higher oxygen availability at BS2, drives sub-phylum-level niche differentiation.

### 4.3. Mineral-Associated Microhabitats and Secondary Sulfur-Bearing Material

The structural and mineralogical heterogeneity of the BS1 sediment establishes a complex biogeochemical matrix that strongly shapes local microbial niches. The primary quartz-clay framework, supplemented by secondary jarosite and gypsum, reflects an environment subjected to intense, localized iron-sulfur cycling under transiently acidic and oxidizing conditions [28,29,30]. Rather than acting as an inert substrate, these secondary sulfate minerals create acidic micro-sites that exert stringent chemical selection, favoring acid-tolerant, sulfur-metabolizing lineages such as *Pyrinomonas*. Simultaneously, the expansive reactive surface area provided by the clay-bearing matrix functions as a critical biophysical buffer, stabilizing local pH-redox microenvironments at the high-temperature venting boundary [31,32]. Beyond Chloroflexota, the contrasting distributions of other abundant bacterial phyla further indicate microhabitat differentiation. Proteobacteria and Bacteroidota were substantially more abundant in BS2 than in BS1, whereas Acidobacteriota showed the opposite pattern, with a markedly higher relative abundance in BS1. This distribution is consistent with ecological differentiation between the hotter, more acidic and sulfur-rich fracture and the cooler, more alkaline adjacent sediment. The higher abundance of Proteobacteria and Bacteroidota in BS2 may reflect a comparatively less thermally stressful environment that permits a broader range of heterotrophic bacterial taxa, whereas the higher proportion of Acidobacteriota in BS1 is consistent with persistence under the more chemically stressed vent-associated conditions. In contrast, Actinobacteriota showed very similar relative abundances in BS1 and BS2 (12.23% and 12.36%, respectively), suggesting that this group was not a major discriminator between the two microhabitats.

In unburned, water-saturated coal seams, the indigenous microbiome is dominated by mesophilic, anaerobic fermenters and methanogens—members of the Firmicutes (*Clostridium, Bacillus*), Proteobacteria and Actinobacteriota, together with hydrogenotrophic and acetoclastic methanogenic archaea—that slowly depolymerize coal macromolecules into alcohols and volatile fatty acids, which are then routed through syntrophic associations into biogenic methane [33]. Combustion inverts this baseline: the heated venting zone exceeds the tolerance of most mesophilic fermenters and methanogens and instead selects for spore-forming thermophiles able to use the reduced combustion gases (H_2_, CO) as electron donors. Several coal-fire metagenomic studies have reported aerobic and anaerobic chemolithoautotrophic Firmicutes (e.g., *Kyrpidia*, *Hydrogenibacillus*, *Brockia*, *Candidatus Carbobacillus*) that oxidize H_2_ and CO, alongside thermophilic Chloroflexota such as Ktedonobacteria and *Thermogemmatispora* [4,23,34]. The Kuitun communities follow the same logic: the Chloroflexota-dominated, thermophilic assemblage at 64 °C (BS1) is consistent with a combustion-driven, gas-oxidizing chemolithotrophic regime, whereas the cooler BS2 sediment retains a more diverse, heterotrophic, soil- and coal-derived Proteobacteria–Bacteroidota flora closer to the temperate fermentative–heterotrophic baseline. Combustion thus does not simply lower diversity; it converts the system from a low-temperature, anaerobic fermentation–methanogenesis food web into a high-temperature, gas-driven chemolithoautotrophic one.

At the BS1 fracture, a macroscopically visible white film was attached to the mineral matrix (Figure 1D). SEM examination revealed wrinkled-to-reticulate surface textures (Figure 4A,C), while EDS of the corresponding region showed a strongly C/O-rich composition, with 76.65 wt% C and 23.35 wt% O and negligible Al, Si, Fe, and S (Figure 4B). The combination of field appearance, film-like morphology, and elemental composition supports the presence of an organic-rich surface film and distinguishes it from the sulfate-rich mineral efflorescences reported from other coal-fire vents [4]. Because the sequenced material was enriched for this film, the BS1 assemblage, in which thermophilic Chloroflexota including Ktedonobacteria, Anaerolineae, and *Thermogemmatispora* were abundant, provides microbial compositional context for this organic-film-bearing mineral surface. Similar film-like material was also observed in the cooler sediment, although it was less developed than at the active fracture, suggesting that organic-film formation extends beyond the hottest microhabitat but is particularly pronounced at the fracture surface. This spatial pattern, together with the enrichment of thermophilic Chloroflexota at BS1, is consistent with strong thermal and geochemical control on the development of this surface-associated microhabitat. In particular, *T. carboxidivorans* has been isolated from a geothermal biofilm and is capable of carbon monoxide oxidation [24], providing a plausible functional link between the thermophilic community and the chemically active fracture environment. Because DNA was extracted from the whole-rock sample containing both the surface film and its underlying sediment matrix, the BS1 community should be interpreted as the integrated microbial assemblage associated with this film-bearing mineral microhabitat rather than as a population confined exclusively to the film. Collectively, these observations identify the active fracture as a heterogeneous, surface-associated microhabitat in which organic films provide an important substrate for microbial colonization and are associated with a thermophile-enriched community.

### 4.4. Comparison with Other Coal-Fire Ecosystems and High-Temperature Environments

Previous coal-fire studies from Centralia, the Kuznetsk Basin, the Altai Mountains, and Xinjiang have documented strong microbial responses to persistent thermal and geochemical disturbance [4,7,23,26,34]. Although thermophilic and stress-tolerant lineages recur across these systems, the dominant taxa and patterns of community response differ among sites. Some environments are characterized primarily by reduced diversity under intense thermal stress and subsequent recovery as temperatures decline [7], whereas others show strong enrichment of specific thermophilic or gas-associated lineages, such as Ktedonobacteria in the Kuznetsk vent or thermophilic Firmicutes in sulfur-rich burning seams [4,5]. These differences may reflect variation in local thermal intensity, gas availability, sulfur chemistry, oxygen exposure, and ecological stage. Thus, coal-fire disturbance appears to impose a common environmental filter, while local physicochemical conditions determine which thermophilic lineages are ultimately favored.

Compared with previous coal-fire studies, the Kuitun site provides a characterization of a post-control residual vent in which strong thermal and geochemical gradients remain expressed over centimeter-scale distances. The paired BS1 and BS2 microhabitats show clear differences in bacterial diversity and in the distribution of thermophilic Chloroflexota, while SEM-EDS and XRD further demonstrate that the hot fracture contains a distinct mineral-organic interface. The recurrence of Ktedonobacteria and related Chloroflexota at Kuitun and in the Kuznetsk vent [4] suggests a shared thermophilic component of coal-fire vent communities, whereas the contrasting assemblage reported from another Xinjiang site [35] highlights substantial site-specific variation. Because these previous studies used different sequencing approaches, cross-site comparisons are interpreted qualitatively rather than quantitatively.

The post-control status of the Kuitun vent may represent an intermediate environmental state between active combustion and ecosystem recovery. However, because the present study is based on a single sampling event, it does not directly resolve temporal succession. The observed Chloroflexota-dominated assemblage should therefore be interpreted as a snapshot of a residual vent state rather than direct evidence of an ecological trajectory.

One fundamental frontier remains unresolved: all empirical observations to date are confined to the surface skin of a much deeper, unsampled subterranean system [4,7,23,34]. At the combustion core, meters below the surface, temperatures exceed 200 °C and surpass the upper thermal limit for life (about 122 °C) [36], rendering the central burning seam abiotic. However, the steep thermal and redox clines radiating outward from this heat engine establish a continuous subterranean transition zone. Environmental filtering frameworks applied to this black box reveal a vertically organized biosphere. Moving upward from an ultra-low-diversity subterranean zone dominated by hyperthermophilic, chemolithoautotrophic archaea and bacteria [2,37], the system crosses the vent-to-sediment interface. This interface is a pivotal ecological tipping zone, where sharp thermal drops and atmospheric oxygen ingress collapse the strict subsurface filter. It acts as the primary gateway, transitioning extreme combustion-driven specialization into rich, soil-derived community assembly. Resolving this three-dimensional ecological continuum will ultimately require deep directional drilling, coupled with downhole sensing and cultivation-independent approaches, beginning with metagenomics [36,37].

### 4.5. Ecological Assembly Mechanisms and Environmental Filtering Dynamics

The observed differences between the two microhabitats are consistent with a combination of environmental filtering and spatially structured dispersal [23,38]. Continuous gas advection at BS1 forms a strong physical and chemical barrier. It likely generates positive gas pressure that restricts atmospheric oxygen infiltration, while imposing severe thermal, acidic and sulfur stress. This convective regime may impose a strong environmental filter. It also limits the passive dispersal of surrounding mesophilic taxa into the active fracture, effectively isolating the vent as a high-temperature microbial island [23]. Toward BS2, gas shear abates, thermal stress weakens, and oxygen enters passively. This relaxation of the environmental filter permits stochastic colonization and soil-derived assemblages to establish, consistent with the increasing importance of stochastic assembly once deterministic stress is relaxed [39].

This dynamic interface is compatible with microbial seed-bank and priority-effect paradigms [40,41,42,43,44]. In fluctuating coal-fire environments, thermal flux and gas emissions vary stochastically, and dormancy becomes a crucial survival strategy [40,41]. Specialized thermophiles and spore-forming lineages likely persist within the mineral matrix as a low-abundance seed bank during low-activity intervals. When high-temperature venting resumes, they germinate rapidly and assert priority effects [41,42]. Reactive mineral surfaces and the carbon-rich, biofilm-like organic films may further provide localized physicochemical refugia for surface-associated microorganisms [45,46].

**Figure 5:**
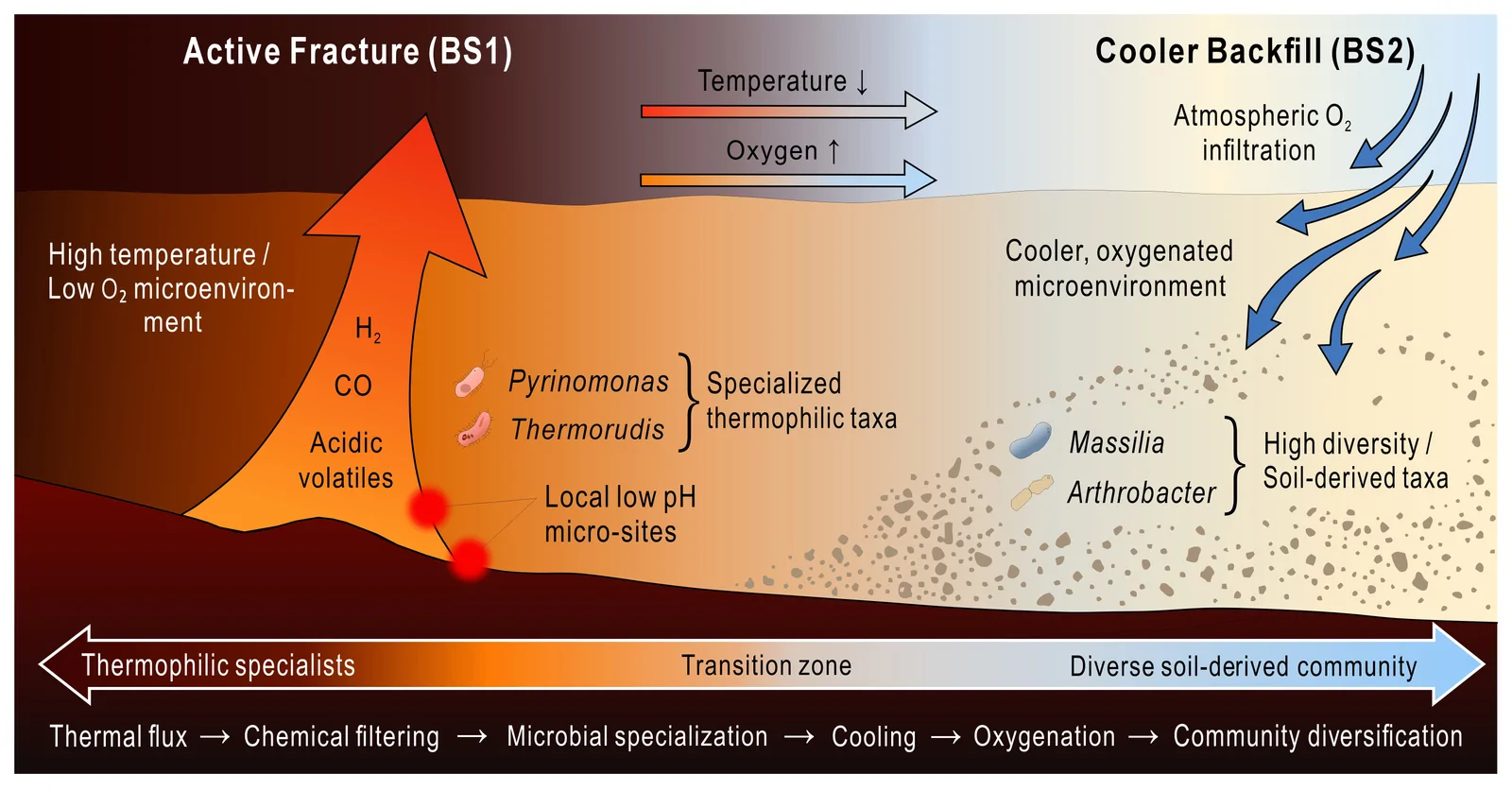
Conceptual model of environmental partitioning and bacterial community differentiation across the sampled coal-fire vent. Temperature, pH and total sulfur are measured values for BS1 and BS2. Arrows indicate hypothesized gradient directions, and taxon labels summarize the observed relative-abundance patterns. Reduced-gas labels (CO, H_2_) and “acidic volatiles” are inferred from previous coal-fire studies and were not measured at the Kuitun vent.

Together, these physical, chemical, and biological factors define an integrated model of coal-fire vent ecology (Figure 5), in which temperature, pH, sulfur speciation, oxygen availability, and mineral microhabitats jointly structure microbial communities along a sharp thermal–geochemical gradient. Comparison of the two adjacent microhabitats provides a high-resolution snapshot of this community differentiation, while the 16S rRNA gene profiles establish a taxonomic framework for future functional investigations. Metagenomic or metatranscriptomic analyses combined with replicated spatial and geochemical measurements will be needed to resolve the metabolic processes and cell–mineral interactions underlying these patterns [47].

## 5. Conclusions

This study demonstrates that bacterial community structure at a residual Xinjiang coal-fire vent is shaped by coupled thermal, geochemical, and mineralogical gradients. The hot gas-emitting fracture (BS1: 64 °C, pH 6.8, total sulfur 5.86 g/kg) supported a lower-diversity community dominated by Chloroflexota, whereas the adjacent cooler sediment (BS2: 43 °C, pH 8.4, total sulfur 1.90 g/kg) harbored a richer community enriched in Proteobacteria and Bacteroidota. Mineralogical and microstructural analyses further revealed a heterogeneous substrate composed of quartz-rich, clay-bearing sediment with secondary sulfate and iron–sulfur-rich domains. Together, these results show that interacting thermal, geochemical, and mineral microhabitats act as ecological filters that structure bacterial communities in non-geothermal coal-fire environments. Future investigations combining replicated spatial sampling, in situ geochemical measurements, and functional omics are needed to determine the metabolic processes underlying these community patterns and to assess whether microbial activity extends into deeper combustion-affected environments.

## Data Availability

The raw sequencing data generated in this study have been deposited in the NCBI Short Read Archive (SRA) database under the BioProject accession number PRJNA1518166 (https://www.ncbi.nlm.nih.gov/sra/PRJNA1518166, accessed on 8 October 2026).
